# Prediction of the Compressive Strength of Recycled Aggregate Concrete Based on Artificial Neural Network

**DOI:** 10.3390/ma14143921

**Published:** 2021-07-14

**Authors:** Liangtao Bu, Guoqiang Du, Qi Hou

**Affiliations:** 1 School of Civil Engineering, Hunan University, Changsha 410082, China; 2 Hunan Hongli Civil Engineering Inspection and Testing Co., Ltd., Changsha 410299, China; houqi19901003@yeah.net

**Keywords:** recycled aggregate concrete, artificial neural network, compressive strength, mixture ratio, sensitivity analysis

## Abstract

Recycled aggregate concrete (RAC), due to its high porosity and the residual cement and mortar on its surface, exhibits weaker strength than common concrete. To guarantee the safe use of RAC, a compressive strength prediction model based on artificial neural network (ANN) was built in this paper, which can be applied to predict the RAC compressive strength for 28 days. A data set containing 88 data points was obtained by relative tests with different mix proportion designs. The data set was used to develop an ANN, whose optimal structure was determined using the trial-and-error method by taking cement content (C), sand content (S), natural coarse aggregate content (NCA), recycled coarse aggregate content (RCA), water content (W), water–colloid ratio (WCR), sand content rate (SR), and replacement rate of recycled aggregate (RRCA) as input parameters. On the basis of different numbers of hidden layers, numbers of hidden layer neurons, and transfer functions, a total of 840 different back propagation neural network (BPNN) models were developed using MATLAB software, which were then sorted according to the correlation coefficient R^2^. In addition, the optimal BPNN structure was finally determined to be 8–12–8–1. For the training set, the correlation coefficient R^2^ = 0.97233 and RMSE = 2.01, and for the testing set, the correlation coefficient R^2^ = 0.96650 and RMSE = 2.42. The model prediction deviations of the two were both less than 15%, and the results show that the ANN achieved pretty accurate prediction on the compressive strength of RAC. Finally, a sensitivity analysis was carried out, through which the impact of the input parameters on the predicted compressive strength of the RAC was obtained.

## 1. Introduction

As one of the most widely used construction materials, concrete consumes as much as 10 billion tons of natural aggregates on the planet every year. China produces 8 billion tons of construction wastes on average every year [1]. The demolition and reconstructing of buildings produces huge amount of construction waste, which further negatively affect the environment. As a matter of fact, lots of countries in the world lack sufficient land to dispose of construction waste. Even countries with comparatively vast territories, like China, face the same difficulty. Without proper treatment, construction waste can result in adverse impacts on environment [2]. To achieve sustainable development and protect the ecological environment that we live in, people have been seeking a new environmentally protective ways of producing concrete for the construction industry [3]. Research on recycled aggregate concrete (RAC) started towards the end of last century [4,5]. Many scholars have studied ways of making concrete using recycled aggregate (RA), based on which, over 75% of construction waste could be reused when making concrete, thereby reducing CO_2_ emissions by a huge amount [6,7,8,9,10,11,12,13,14].

However, due to the powerful absorption performance of RA and the poor adhesion performance between RA and the cementing material, both the compressive strength and the elastic modulus of the RAC are reduced [15]. Many scholars have studied the factors influencing the compressive strength of RAC, and these mainly include: water content, replacement rate of recycled aggregate, and water–cement ratio [16,17]. In 1993, Merlet et al. [18] proposed a new concrete mixed with waste materials for the first time, and studied its performance when adopting different proportions of fine-grained waste concrete. Heidari A et al. [19] studied concrete production using waste bricks and conducted tests for compressive strength and bending strength. Tavakoli et al. [20] studied the replacement of sand in concrete by clay bricks, and the effects of concrete having its sand substituted by different ratios of clay bricks. They figured out the optimal clay brick substitution rate, and finally found no significant changes in concrete performance.

Concrete is the most basic building material. Its quality can seriously affect the safety of the structure, so that both the construction units and the quality inspection departments attach great importance to the compressive strength of concrete. The traditional method of testing the compressive strength of concrete is to reserve a test specimen, which is complicated to carry out. To better detect the compressive strength of concrete, some scholars have studied prediction models for concrete compressive strength [21,22]. The basic properties of RAC must be verified by practical experiments, because concrete performance can be greatly affected by the composite material types and the amount of use. However, lab experiments usually require a great amount of manpower, materials, and funds. In this case, a probability model could be adopted to predict the concrete performance. However, when there is a great number of variables and complicated relations between independent variables and dependent variables, the probability model is no longer applicable [23]. Since RAC is mixed with a large amount of recycled materials and very complicated components, it is hard to accurately predict its performance using traditional regression prediction approaches [24,25,26].

Thanks to the development of information technology, artificial intelligence, big data and other means have been extensively applied in engineering areas. During recent years, artificial neural network (ANN), an artificial intelligence algorithm inspired by nature, has been widely used in the modeling field for practical problems. ANN can perceive complex nonlinear relationships between dependent variables and independent variables, and effectively solve many complex engineering problems. It has been widely used in civil engineering, such as groundwater monitoring, structure recognition, structural damage monitoring, traffic engineering, material behavior modeling and foundation settlement prediction, etc. Wagh et al. [27] used an ANN model to detect irrigation use of groundwater, and showed excellent performance with 13 physical and chemical characteristics as input parameters. Deshpande et al. [28] predicted the compressive strength of concrete by means of ANN, model tree and nonlinear regression, and the results indicated that the ANN model provided the highest accuracy. Xiong et al. [29] obtained structural images after geological disasters using unmanned aerial vehicles by virtue of ANN image recognition technology, and judged whether regional structures had collapsed using this technology, and evaluated the damage after disasters on the basis of structural appearance characteristics. Lv, Y et al. [30] came up with a model based on a BP neural network and grey theory to predict the settlement of a foundation pit. The results suggested that both models boasted favorable predictive capacity. Lots of scholars have used ANN to predict concrete performance [31,32,33,34,35]. Due to the powerful learning ability of ANN, some scholars have tried to predict concrete performance using ANN by taking the material components of the concrete as parameters [36]. Torre et al. [37] constructed a multi-layer perceptron model to accurately predict the compressive strength of high-performance concrete. Topçu et al. [38] used ANN to predict the compressive strength and the splitting tensile strength of recycled aggregate concrete containing silica fume. Khademi et al. [39] adopted three artificial technologies—ANN, ANFIS and MLR—to predict the compressive strength of RAC, for which the results showed that ANN was able to predict the compressive strength of RAC more accurately than the other two. To predict the compressive strength of the self-compacting high-strength concrete mixed with silica fume, fly ash, and blast furnace slag aggregates, Jamaldin et al. [40] established a neural network model, based on which they obtained good predictions of the experimental results. At present, ANN is mainly used to predict the compressive strength of natural aggregate concrete and concrete containing blast furnace slag and fly ash, but similar research has rarely been performed on RAC due to its complex composition. RAC is a new type of material that is different from traditional concrete in terms of both the concrete components and its performance. It is hard to predict the compressive strength of RAC using the regressive statistical method. ANN has the ability to capture the nonlinear and complex relationships between variables from existing actual data. Therefore, the application of ANN in the prediction of RAC performance is a significant research topic.

To make up the gap of using ANN for predicting the RAC compressive strength and test the compressive strength of RAC in a more efficient manner, in this study, a RAC compressive strength prediction model was established based on an artificial neural network. The training data set was obtained through experiments, which was used to develop the ANN model. Meanwhile, a neural network model with two hidden layers was constructed, which was trained and tested using 88 groups of data that were obtained from experiments. The established neural network model had 8 input parameters and 1 output parameter. The prediction results were compared with the test results, verifying the reliability of the model. Finally, a sensitivity analysis was carried out on the parameters to analyze the influences of the RAC parameters on its compressive strength.

## 2. Experiment Plan

### 2.1. Materials

**Figure 1:**
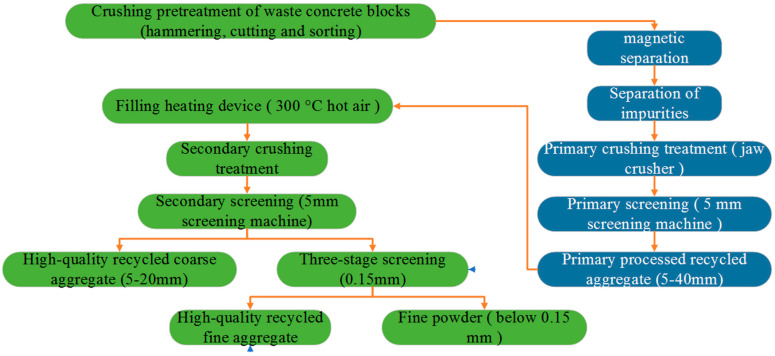
Recycled coarse aggregate content (RCA) production process.

**Figure 2:**
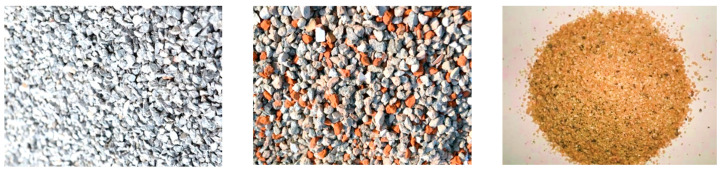
Natural coarse aggregate (NCA), RCA and river sand.

**Figure 3:**
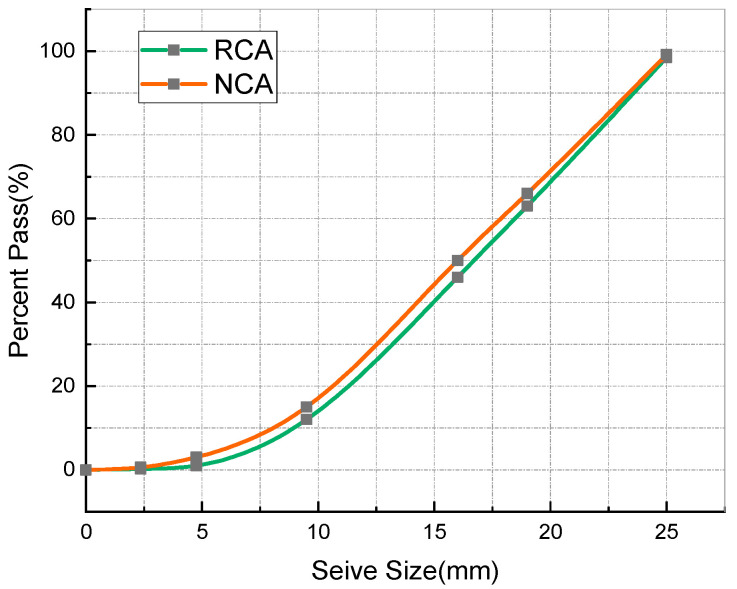
Particle size distribution of RCA and NCA.

**Figure 4:**
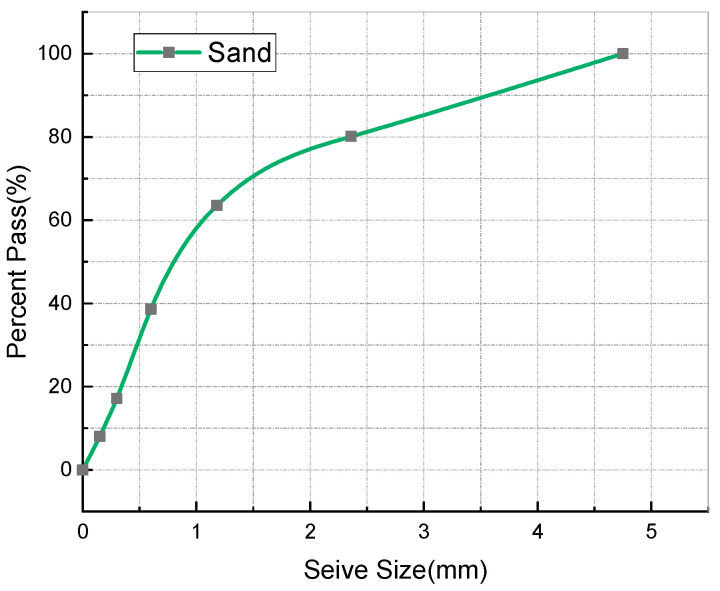
Particle size distribution of river sand.

Portland cement, with chemical and mineral components as shown in Table 1 and physical properties as shown in Table 2, was adopted in the experiments carried out in this study. The waste concrete was provided by Changsha Muck Industry Association. Firstly, its impurities were removed, and then it was crushed using a stone crushing machine to produce RCA. The production process is shown in Figure 1. The recycled aggregates adopted in this study contained 97% concrete aggregate and 3% masonry aggregate, with 0–25 mm continuous gradation and a 16% crushing index. Meanwhile, in the experiments carried out in this paper, the gravel crushed by granite was taken as the natural coarse aggregate (NCA), with a largest particle size of 25 mm and a crushing index of 12%. After being washed with water, the silt content of the NCA reached 0. Natural river sand was used as the fine aggregate, which had a largest particle size of 5 mm. Refer to Figure 2 for the NCA, RCA, and river sand, and refer to Table 3 for the physical properties of the main materials. It can be seen from Table 3 that the RCA had a lower density, but a far greater water absorption rate than the NCA. This is because RCA is porous, and cement mortar is attached to the surface of the aggregate [41]. The grading of the aggregates was determined on the basis of the procedures described in the national standard JGJ52-2006 [42], using test sieves with standard specifications. The gradation results for the RCA, NCA and river sand are as shown in Figure 3 and Figure 4.

### 2.2. Design of Mixing Proportion

**Figure 5:**
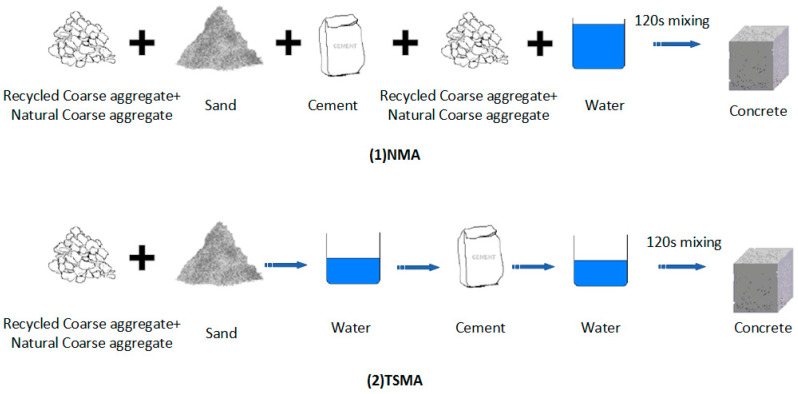
The mixing process of all components.

To better predict the compressive strength of RAC, a total of 88 different concrete mix proportions were designed. It can be ascertained by reviewing the existing literature that the compressive strength of RAC is subject to many factors, mainly including cement content (C), sand content (S), natural coarse aggregate content (NCA), recycled coarse aggregate content (RCA), water content (W), water–colloid ratio (WCR), sand rate (SR), and replacement rate of recycled aggregate (RRCA). According to the above factors, before the experiment, RAC was prepared with different mixing proportions. RRCA was set as 0–100% of the total volume of coarse aggregate at 10% intervals. The experiment was divided into two parts: P1 and P2. Each part contained four groups—G1–G4 and G5–G8—where the sand ratio of G1–G4 was 35%, and the water–cement ratio were respectively 0.5, 0.55, 0.6, and 0.65. As for G5–G8, the sand ratio was 30%, while the water–cement ratios were 0.32, 0.37, 0.42, and 0.47, respectively. RAC is characterized by high porosity, high impurity content, and cement mortar residue on the aggregate surface, all of which seriously affect its mechanical properties. Therefore, higher requirements need to be met during the material mixing stage of RAC. To improve the compressive strength of RAC, a new concrete two-stage mixing approach (TSMA) proposed by Vivian W.Y. Tam et al. [43] was used in this study. Additionally, specimens were made according to the GB/T50081-2002 standard [44]. The normal mixing approach (NMA) is first to add half of the coarse aggregate, then the fine aggregate and cement, and finally the residual coarse aggregate; after that, water is added, and the mixing machine is immediately started [45]. However, the TMSA actually divides the mixing process into two parts and divides the required water into two parts as well, according to specific proportions, to be added at different times. Figure 5 shows the mixing processes for the two different approaches.

### 2.3. Experiment Process

**Figure 6:**
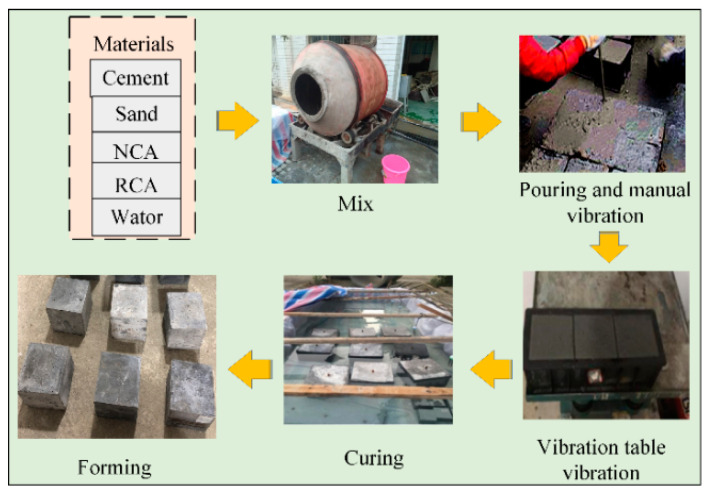
RAC specimen preparation process.

**Figure 7:**
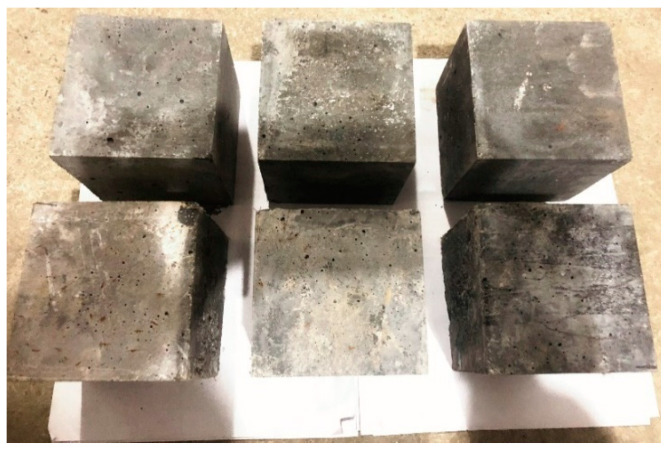
Cubic specimens.

During the process of preparing the RAC specimens, a JZC forced drum mixer was used for mixing. In addition, vibration was applied using a vibrator, and manual tamping was conducted. Figure 6 shows the RAC specimen preparation process. Step 1: put the aggregate and half of the water into the mixer to mix for 2 min, then, put the remained water and materials into the mixer and mix for 2 more minutes; Step 2: pour the concrete into the mold and tamp it manually, after that, vibrate it using a vibrator for 3 min; Step 3: 24 h after pouring, demold the specimen, and cure for 28 days in the curing room at a temperature of 20 ± 2 °C and 95% relative humidity. A total of 88 different RAC mix proportions were prepared under the same conditions. A total of 3 samples were made for each mix proportion. Each specimen was made with a size of 150 mm × 150 mm × 150 mm. Please refer to Figure 7. After that, in order to test the workability of concrete, a concrete slump test was carried out according to the JGJ52-2006 standard specifications [42].

**Figure 8:**
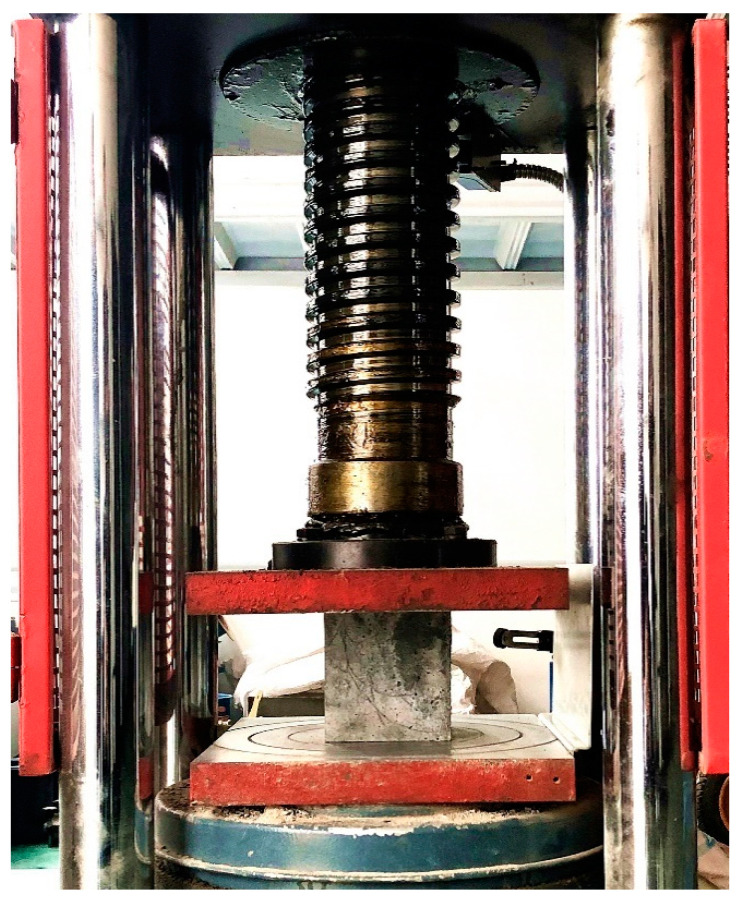
Cube compressive strength test.

**Figure 9:**
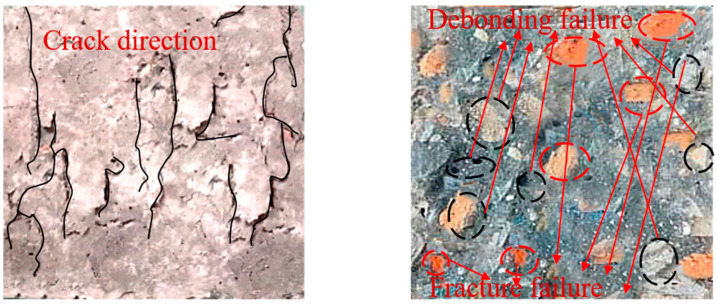
Specimen failure crack and failure interface morphology.

Finally, in accordance with the GB/T50081-2002 [44] standard, a cube compressive strength experiment was carried out using a TYA-2000 (Shenzhen wance Test Equipment Co., Ltd., Shenzhen, China) electro-hydraulic compressive tester; the test process is shown in Figure 8. The specimen failure crack and failure interface morphology are presented in Figure 9. It can be seen that the cracks on the RAC surface are mostly vertical. The part highlighted in red in the figure indicates the fracture failure of the aggregate after specimen fracture, while the part highlighted in black indicates the peeling failure of aggregate and mortar. This proves that while the natural aggregate usually exhibits peeling failure between the aggregate and the mortar, the recycled aggregate mostly exhibits fracture failure of aggregate itself. This is also one of the reasons causing the low compressive strength of RAC. Therefore, the bending strength of the recycled aggregate is highly important to the compressive strength of RAC.

### 2.4. Experimental Results

Throughout the experiment, a data set containing 88 different mixing proportions was obtained, which was used to train the ANN model. Table 4 shows all the experimental mixing proportion data.

**Figure 10:**
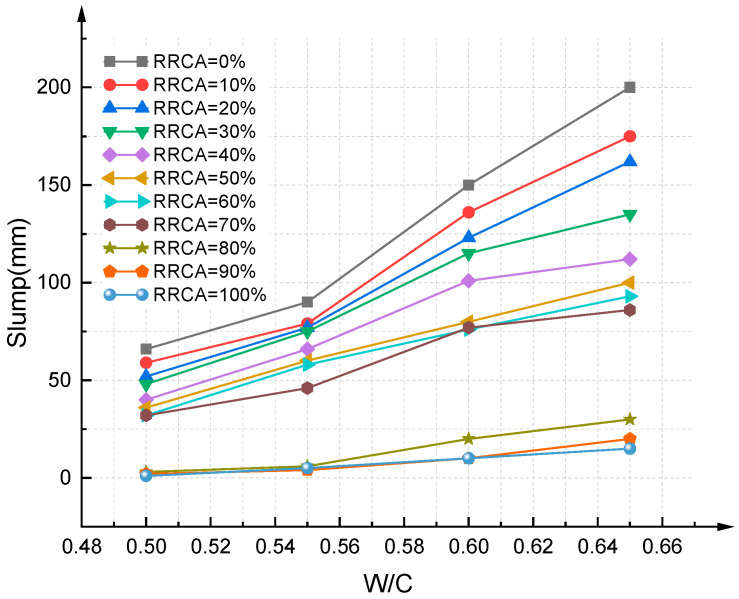
Effect of water–cement ratio on slump (P1).

**Figure 11:**
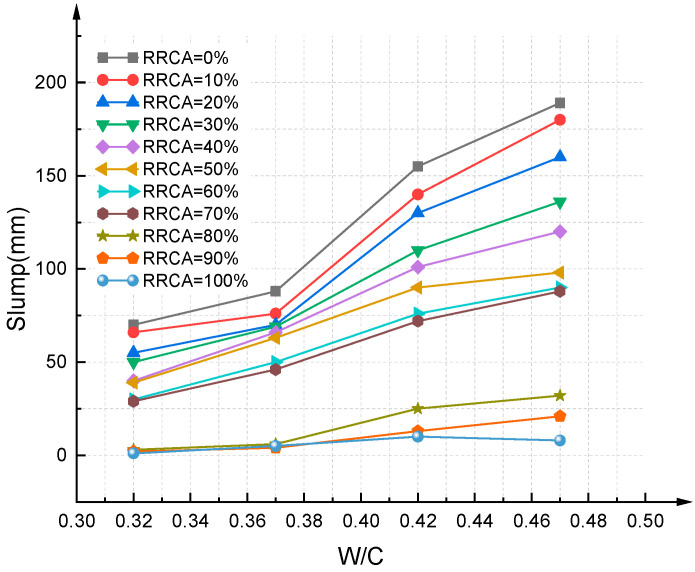
Effect of water–cement ratio on slump (P2).

On the basis of the slump experiment, the effect of the water–colloid ratio on the slump was obtained; see Figure 10 and Figure 11. The results show that the RAC slump increases with increasing water–cement ratio, which is similar to ordinary concrete. When the water–cement ratio is constant, the slump decreases with increasing RRCA. Because RCA has greater water absorption property compared to NCA, under the same water–cement ratio conditions, the higher the RRCA, the worse the workability and the lower the slump of the RAC. This is a rule concluded under the premise of guaranteeing a dry state of RCA and actual water–colloid ratio, which is in line with the conclusions obtained by [46,47].

**Figure 12:**
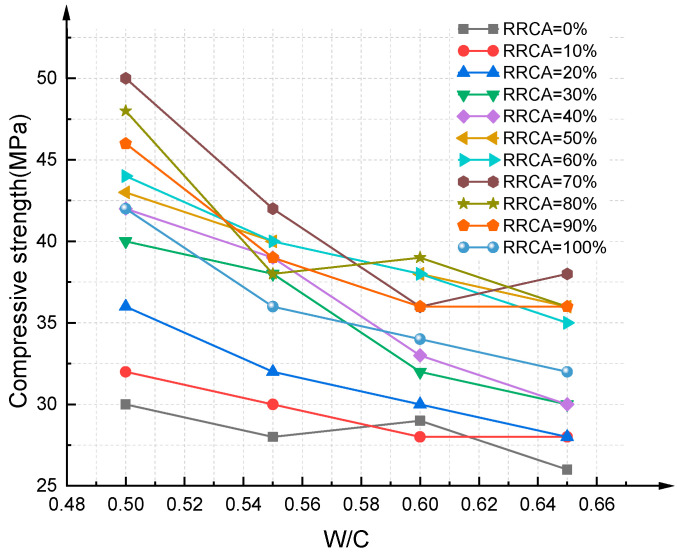
Compressive strength experiment results of RAC cube specimens (P1).

**Figure 13:**
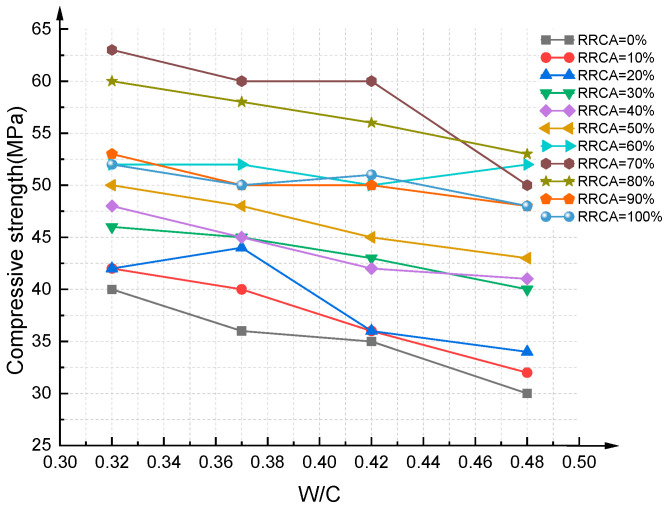
Compressive strength experiment results of RAC cube specimens (P2).

Figure 12 and Figure 13 show the results of the compressive strength experiments of the RAC cubic specimens with 88 different mixing proportions. The mean value of three specimens was taken as the final compressive strength. It can be seen from the figure that when the RRCA is 70%, the compressive strength of RAC reaches its maximum value; the compressive strength of RAC using the best mixing proportions reached as high as 63 MPa, which is equivalent to that of ordinary concrete.

## 3. Strength Prediction Model

### 3.1. Artificial Neural Network

**Figure 14:**
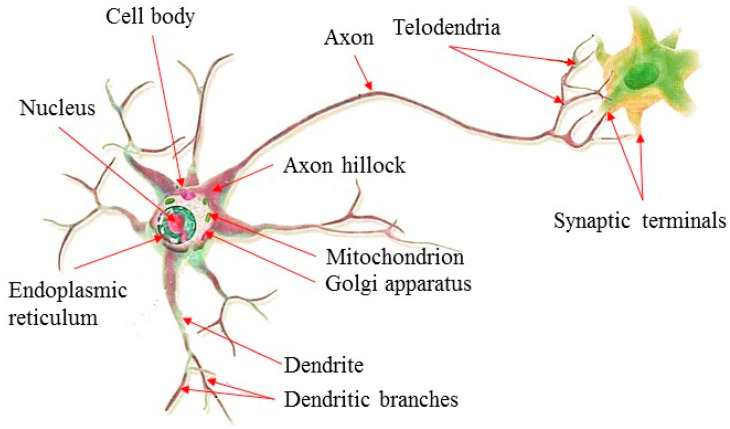
Schematic diagram of biological neuron structure.

ANN is composed of many interconnected neurons, each of which is capable of information processing [48]. It is actually a complex mathematical model, which simulates the biological neuron structure and self-learning function; see Figure 14. As a matter of fact, ANN is quite capable of simulating the human brain, and is able to process information and make corresponding predictions [49,50]. ANN is able to learn the relationships between the input and the output through a mathematical training process, thus reducing errors and achieving optimal prediction. Its most outstanding features are the ability to learn from existing data, in order to classify and predict data, and to assist in making decisions. Based on relative training, ANN is able to map the input parameters to the specific output. Compared with traditional numerical value analysis, ANN achieves more reliable prediction results [51,52,53,54]. The multi-layer feedforward neural network usually has an input layer and an output layer, as well as multiple hidden layers. Among the existing training algorithms, the error backpropagation algorithm is able to achieve the most satisfactory results. It can continuously update the weights and thresholds of the network according to the known errors until the minimum error of the network is reached.

### 3.2. Back Propagation Neural Network

**Figure 15:**
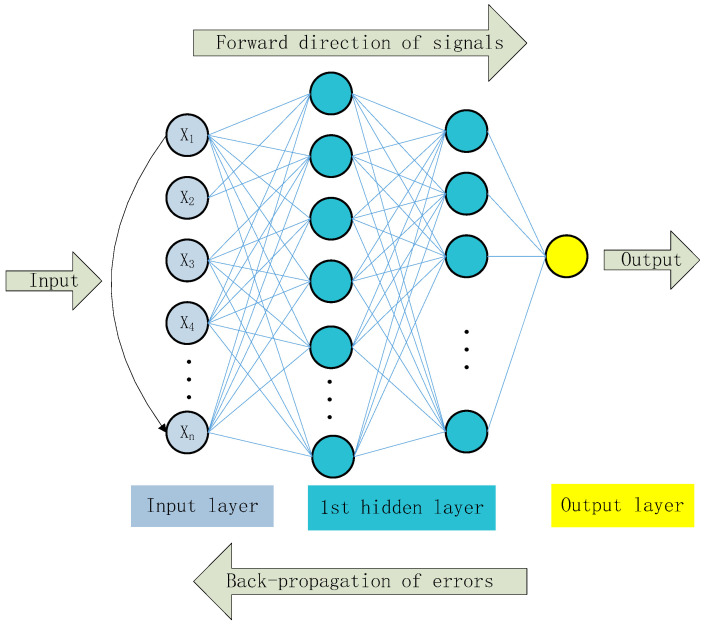
Schematic diagram of the BPNN structure of a multi-layer perceptron.

**Figure 16:**
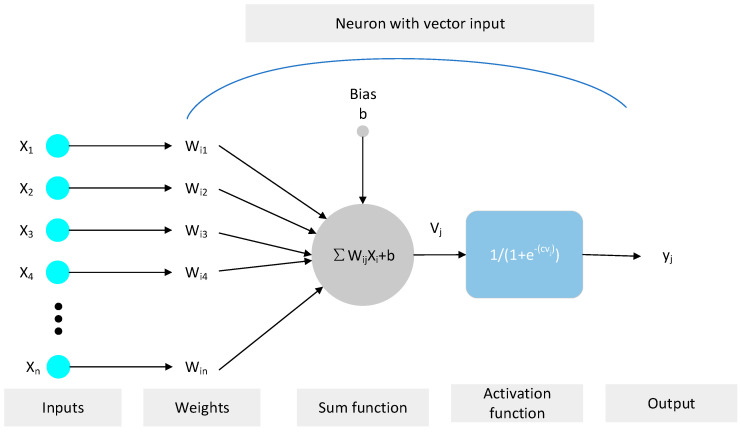
Information processing process of a single hidden layer neuron.

Back propagation (BP) is a learning algorithm developed by Rumelhart et al. [55], and is most commonly used in perceptron networks with hidden units. Meanwhile, the BPNN is also an ANN structure that nowadays finds wide application. The BP algorithm mainly includes two processes: first, the input signal is transmitted from the input layer to the output layer, and then the output result â is compared to the target value a. The error of each neuron is determined on the basis of the difference between the predicted value and the target value, which is the back propagation process of error. Second, the weight and threshold between the predicted value and the target value should be readjusted to reduce the error between the predicted value and the target values. According to the generalized delta principle, iterative training is performed through the gradient descent method until minimal error between the predicted value and the target value is obtained, that is, the loss function reaches its minimum value. On the other hand, the multi-layer perceptron is a more complicated perceptron, and is the most widely used [56,57,58]. It contains an input layer, multiple hidden layers, and an output layer. Please see Figure 15 for the BPNN structure of the multi-layer perceptron. Figure 16 shows the information processing of the single hidden layer neuron. Each neuron needs to cover the input, weight, threshold, and activation functions. In addition, the process of adjusting the weight to produce the target output is actually the “training” [59]. X_i_ = (X_1_, X_2_, X_3_… X_n_) represents the input parameters of BPNN, while W_ij_ = (W_i1_, W_i2_… W_in_) represents the corresponding weight of each input. Formula (1) is the summation function formula. Formulas (2) and (3), respectively, represent the updated weight and threshold.

(1) $$V_{j}=\sum_{i=1}^{n}W_{\mathrm{ij}}X_{i}+b$$

(2) $$w(k+1)=w(k)-\alpha \frac{\partial E(k)}{\partial w(k)}$$

(3) $$b(k+1)=b(k)-\alpha \frac{\partial E(k)}{\partial b(k)}$$

where α is the learning rate, for which the value is set to be 0.01; w(k) and b(k) are the connection weights and threshold vectors among layers at the kth iteration; $\frac{\partial E(k)}{\partial w(k)}$ and $\frac{\partial E(k)}{\partial b(k)}$ are the error adjustment gradients of the output errors to each weight and threshold at the kth iteration.

### 3.3. Transfer Function

The transfer function has a great impact on the performance of the neural network. It can add nonlinear factors to the model, thereby enhancing the expression of the model. The commonly used activation functions mainly include the sigmoid function, tanh function, and ReLU function. Previous studies have shown that the S function is the optimal transfer function [60,61]. Therefore, in this study, the sigmoid transfer function (Log-sigmoid, tan-sigmoid) was used, as shown in Formulas (4) and (5). All ranges of input data are acceptable to this function, which can further control the output within a range of [0, 1].

(4) $$Log-sigmoid:g(x)=\frac{1}{1+e^{-n}}$$

(5) $$\mathrm{Tan}-sigmoid:g(x)=\frac{2}{1+e^{-2n}}-1$$

### 3.4. Training Algorithm

The BPNN can be trained using many different training algorithms, among which the gradient descent algorithm, Newton algorithm, conjugate gradient algorithm, Cauchy-Newton algorithm and Levenberg-Marquardt algorithm are the most widely used ones. The Levenberg-Marquardt algorithm has been widely applied in ANN prediction, achieving the best prediction for the nonlinear behavior of concrete. However, this is an algorithm quite different from the others [62], and was implemented and improved in MATLAB (MathWorks, r2016A) software.

### 3.5. Data Standardization

Data standardization is a key step in the soft computing process, and can eliminate the influences of different dimensions on the data processing results. In the field of neural networks, the input data are usually scaled to [0, 1]. This not only eliminates the influences of different dimensions of output parameters, but also reduces the size of the input data and speeds up the training process of the neural network [63]. Iruansi et al. [64] also pointed out that data normalization, within an appropriate range, can improve the learning efficiency of neural networks. In addition, in this paper, the data standardization formula is as shown below (6).

(6) $$x_{n}=\frac{x-x_{\min}}{x_{\max}-x_{\min}}$$

where $x_{n}$ is the value after standardization, $x_{\max}$ is the maximum value of para.x, and $x_{\min}$ is the minimum value of para.x.

### 3.6. Model Evaluation Parameters

The BPNN model was trained using the training data, and then its accuracy was evaluated on the basis of the prediction errors obtained using the verification data. Besides calculating the “goodness of fit” of the model, it is also necessary to analyze the error of the model in order to conduct better evaluation of the model. In this study, Formulas (7)–(9) were used to calculate the correlation coefficient (R^2^), mean square error (MSE), and root mean square error (RMSE), respectively.

(7) $$R^{2}=1-\frac{\sum_{i=1}^{n}{\left({CS}_{E}-\overset{}{{CS}_{P}}\right)}^{2}}{\sum_{i=1}^{n}\left({CS}_{E}-{\bar{CS}}_{E}\right)}$$

(8) $$MSE=\frac{1}{n}\sum_{i=1}^{n}({CS}_{P}-{CS}_{E}{)}^{2}$$

(9) $$RMSE=\sqrt{\frac{1}{n}\sum_{i=1}^{n}{\left({CS}_{P}-{CS}_{E}\right)}^{2}}$$

where ${CS}_{P}$ is the predicted output value of the model; ${\bar{CS}}_{E}$ is the mean experimental value; ${CS}_{E}$ is the target output (experimental value); and n is the total number of samples.

The correlation coefficient R^2^ can be used to measure the linear correlation between variables. The closer the R^2^ gets to 1, the stronger the correlation between the variables. MSE and RMSE can be used to evaluate the difference between the predicted value and the target value. The smaller the MSE or RMSE value, the better the accuracy of the approach using the prediction model to describe the experimental data [65].

### 3.7. Determination of BPNN Structure

The first step of model development is to determine the BPNN structure, which should be achieved by figuring out the optimal numbers of hidden layers and hidden layer neurons. Meanwhile, BPNN over-fitting is also a problem that should be considered. The more complicated the model, the greater the possibility of the occurrence of the over-fitting problem. Lots of scholars have proposed relative algorithms that avoid over-fitting [66,67,68]. In this study, a trial-and-error approach was used to determine the optimal BPNN structure, for which the whole process was realized in MATLAB software.

## 4. Discussion

### 4.1. BPNN Architectures

**Figure 17:**
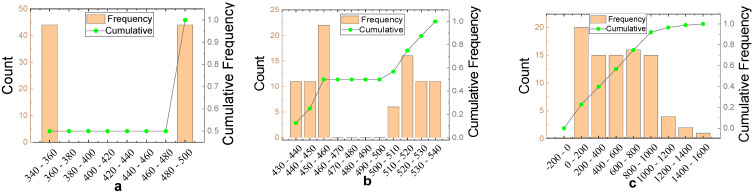

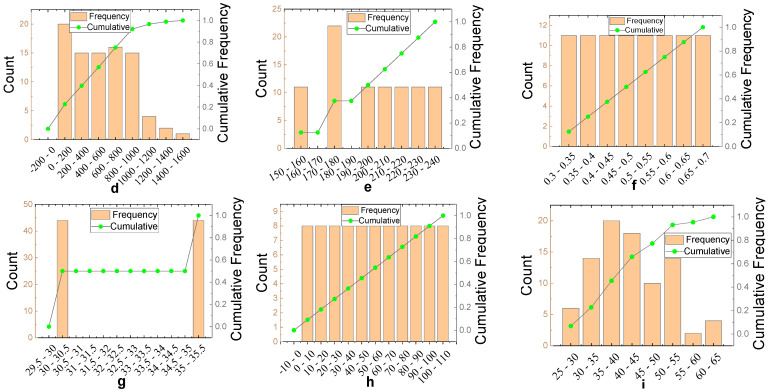
Histogram of variable frequency distribution.{(**a**) C (Kg/m^3^); (**b**) S (Kg/m^3^); (**c**) NCA (Kg/m^3^); (**d**) RCA (Kg/m^3^); (**e**) Water (Kg/m^3^); (**f**) W/C; (**g**) SR (%); (**h**) RRCA (%); (**i**) CS (MPa)}.

The dimension of the input parameter vector is 1 × 8, which consists of eight parameters: C (kg/m^3^), S (kg/m^3^), NCA (kg/m^3^), RCA (kg/m^3^), Water (kg/m^3^), W/C, SR (%), RRCA (%). The output vector dimension is 1 × 1, namely the RAC compressive strength (CS). For the numerical value statistics of these parameters, please see Table 5. In addition, the frequency distribution histogram of the nine variables is as shown in Figure 17. In addition, the training parameters of the BPNN model are as shown in Table 6.

### 4.2. BPNN Model Development

**Figure 18:**
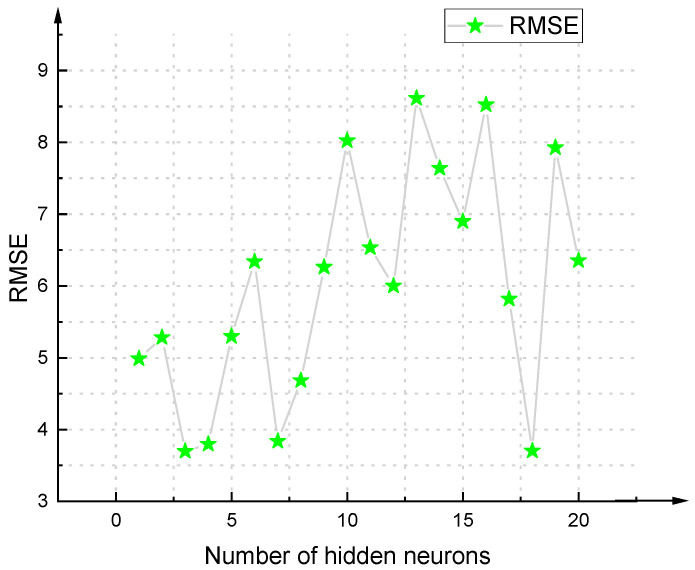
RMSE value of BPNN model based on Log-Sigmoid (single hidden layer).

**Figure 19:**
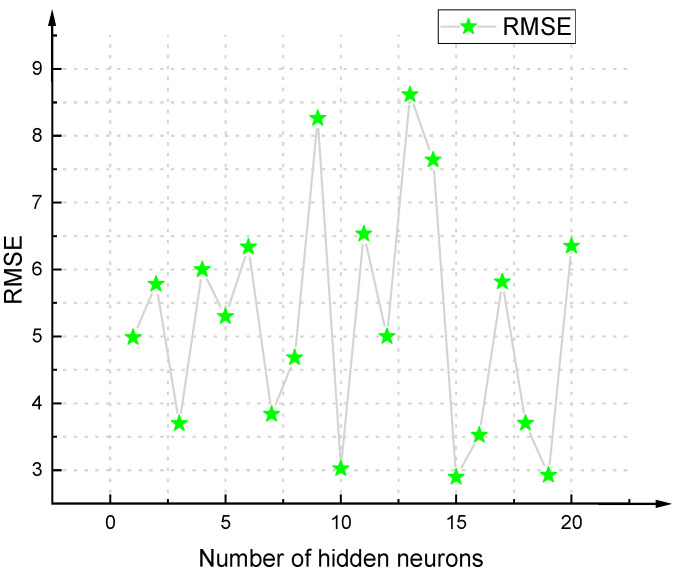
RMSE value of BPNN model based on Tan-Sigmoid (single hidden layer).

**Figure 20:**
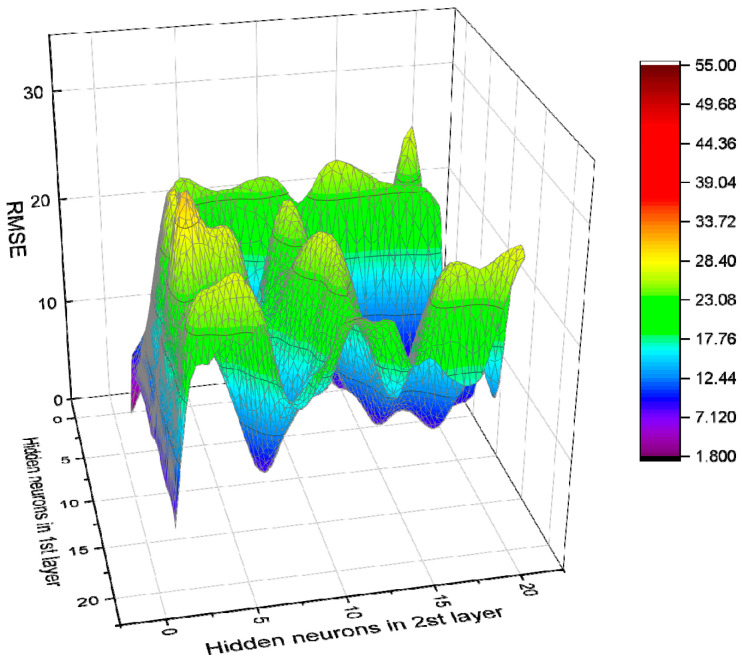
RMSE value of BPNN model based on Log-Sigmoid (double hidden layers).

**Figure 21:**
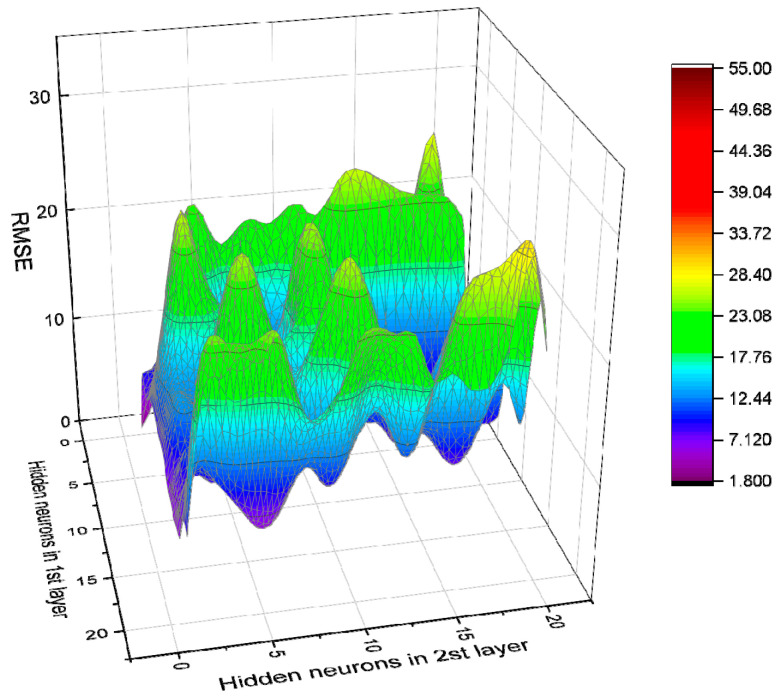
RMSE value of BPNN model based on Tan-Sigmoid (double hidden layers).

There is still no specified theoretical basis for determining the best structure of the BPNN. Most scholars now use a trial-and-error approach to determine it [69,70]. Based on different settings of BPNN parameters, a total of 840 BPNN models were studied and developed. In addition, each model was trained using 62 (70.45%) data, and tested using 26 (29.55%) data, to verify the model. Then the correlation coefficient R^2^ was used to sort the 840 developed BPNNs. Top 10 models in the sorting are as shown in Table 7. As for the RMSE of each model, please see Figure 18, Figure 19, Figure 20 and Figure 21.

**Figure 22:**
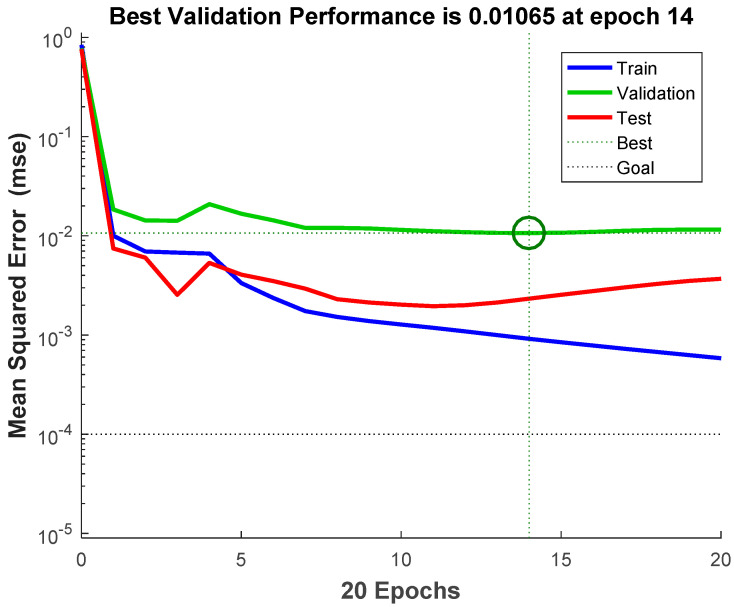
Error changes in the BPNN training process.

**Figure 23:**
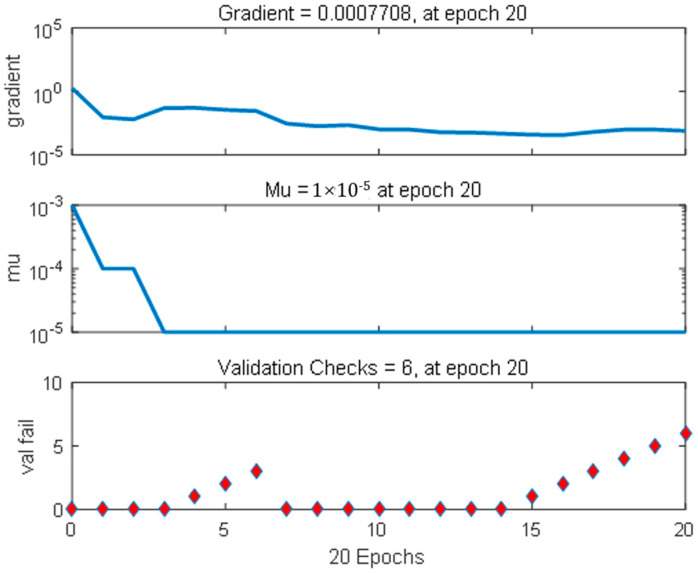
BPNN training status.

Figure 22 shows the error reduction process during the BPNN training, which provides an optimal representation of the model training process. The blue line in the figure represents the network training error, the green line represents the network verification error, and the red line represents the test error. The training stops when the verification error reaches the set target or the verification error is no longer decreasing. Figure 23 shows the training status of BPNN.

## 5. Results

**Figure 24:**
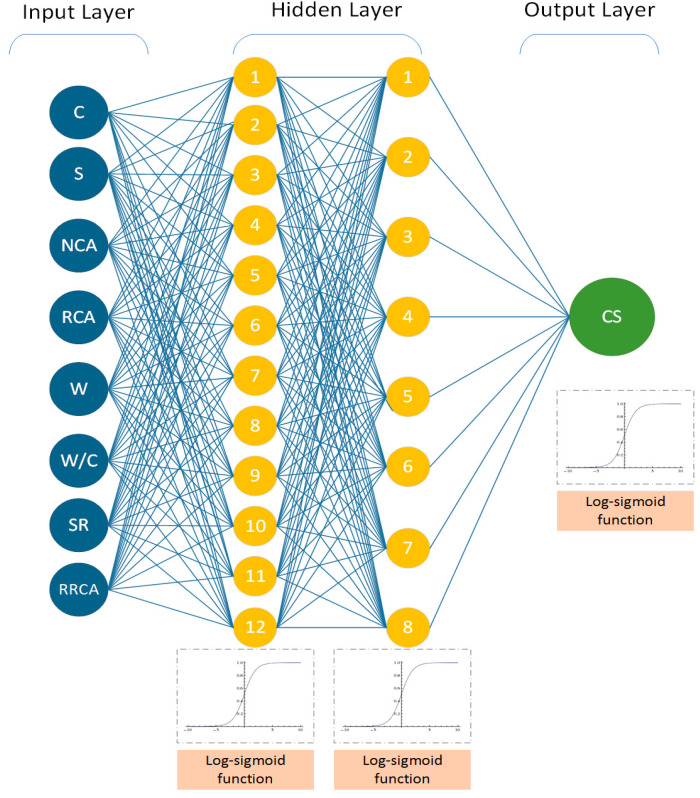
Optimal BPNN structure.

All models were realized using MATLAB software according to the above process. A total of 840 BPNN models were developed based on different numbers of hidden layers, hidden layer neurons, and transfer functions. It can be seen from Table 7 that the optimal BPNN structure was 8–12–8–1, with a correlation coefficient R^2^ of 0.96650; the RMSE was 2.42, and the activation function was the Log-sigmoid function. This structure has two hidden layers: the first hidden layer contains 12 neurons, while the second contains 8 neurons; see Figure 24.

**Figure 25:**
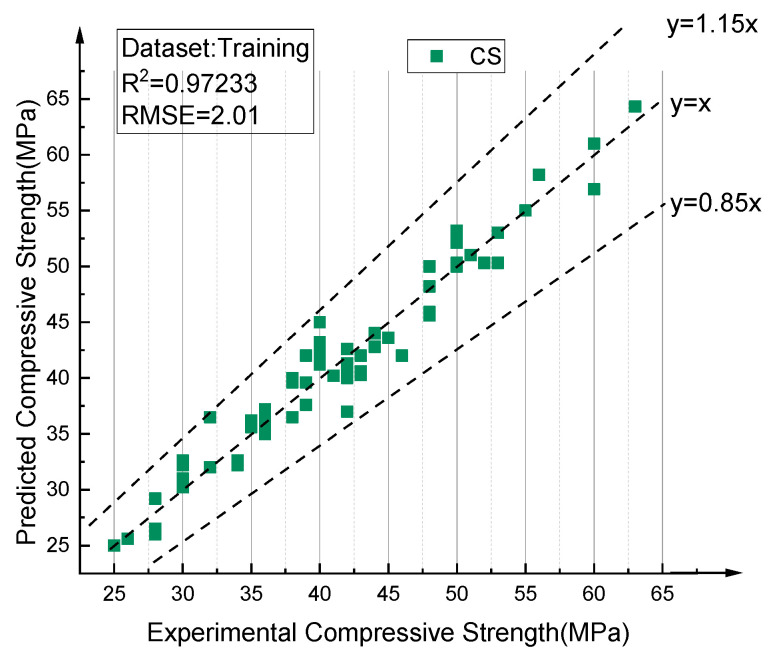
Comparison between prediction values and test values of RAC compressive strength (training data).

**Figure 26:**
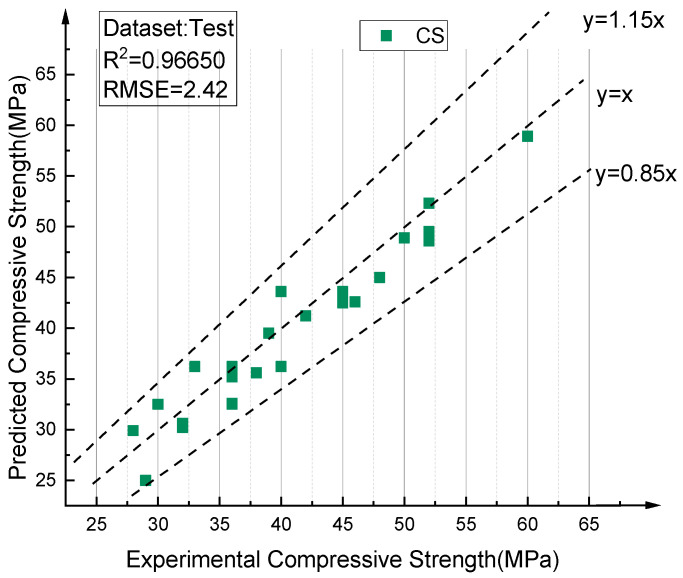
Comparison between prediction values and test values of RAC compressive strength (test data).

**Figure 27:**
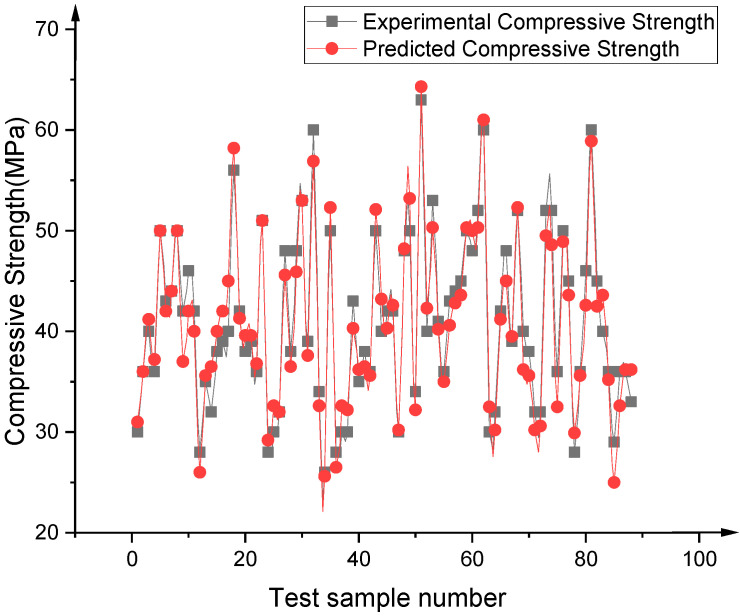
Comparison between prediction values and test values of RAC compressive strength (all data).

Figure 25 and Figure 26 describe the results predicted by the optimal BPNN model, which respectively compares the values predicted by the training set and the test set with the experimental values. Obviously, the proposed 8–12–8–1 BPNN model is capable of making accurate predictions with respect to RAC compressive strength, and controlling the deviations of all samples to within 15% (points between the two dotted lines). Figure 27 shows the comparison between the predicted values of all data and the experimental values, achieving the same conclusions.

## 6. Sensitivity Analysis

In the field of neural networks, many scholars have conducted research on new ANN learning rules, restructuring the architecture of the neural network in order to achieve better application. ANN is called a “black box”, aiming to convert the input into an ideal output. Being different from other traditional numerical analysis models, it is difficult to use ANN to interpret the relations between independent variables and dependent variables. The input parameters contain the required output information, while the remaining additional features and information in the input parameters are beneficial for improving the prediction capability. However, usually, some redundant parameters containing little information are also included, which do not improve the information, and can affect the performance of the learning algorithm. The purpose of the sensitivity analysis is to determine the impact of the input parameters in the mathematical model on the output result, thereby enhancing the understanding of the input and output variable relationships in the model. The sensitivity analysis can be used to determine the contribution of a single input parameter to the output parameter, thus reducing redundant parameters [71]. In this study, a relative analysis was conducted using the sensitivity analysis method based on weight, as proposed by Milne [72]; see Formula (10), below:

(10) $$IIF=\frac{\sum_{j=1}^{n_{hidden}}\frac{w_{ji}}{\sum_{l=1}^{n_{inputs}}\left|w_{jl}\right|}\cdot w_{oj}}{\sum_{k=1}^{n_{inputs}}\left(\sum_{j=1}^{n_{hidden}}\left|\frac{w_{jk}}{\sum_{l=1}^{n_{inputs}}\left|w_{jl}\right|}\cdot w_{oj}\right|\right)}$$

In the formula, IIF is the importance of the input parameters, which are referred to as contributory factors; w is the connection weight between the two connected neurons; $w_{ji}$ is the connection weight between the input layer and the hidden layer, and $w_{oj}$ is the connection weight between the output layer and the hidden layer (product of the weight of the first hidden layer and the weight of the second hidden layer). l,i,k all represent the input layer neuron, $n_{inputs}$ is the number of the input parameters, and $n_{hidden}$ is the number of hidden neurons (first hidden layer).

**Figure 28:**
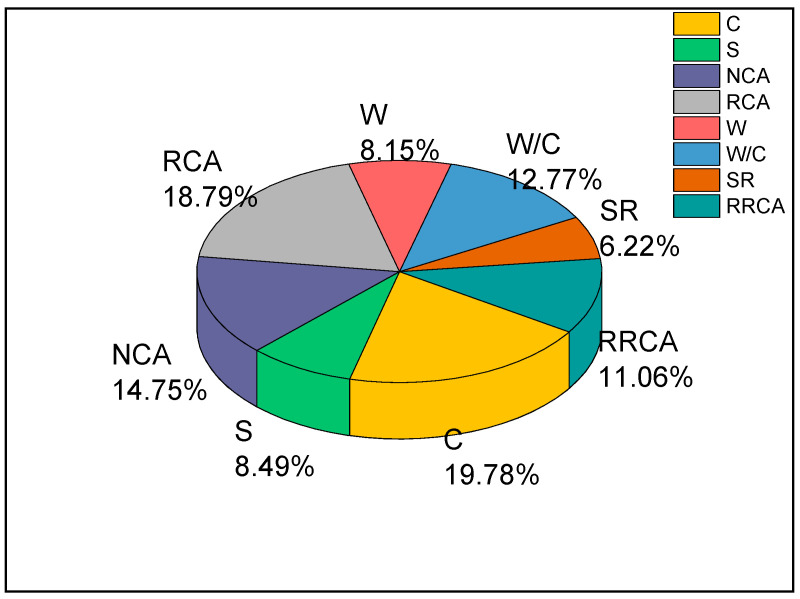
Single parameter effect on the prediction of RAC compressive strength.

Table 8 shows the connection weight between the input layer neurons and the hidden layer neurons of the optimal BPNN model. Figure 28 shows the effect of a single parameter on the prediction of RAC compressive strength. It can be seen that the parameter of cement content has the most significant impact on predicted compressive strength of RAC, with an impact factor reaching 19.78%. This indicates that the cement content is the factor that affected the compressive strength of the recycled concrete the most in this study. The impact factors of RCA, NCA, and W/C were, respectively, 18.79%, 14.75%, and 12.77%, indicating that the content of the aggregate has a greater impact on the compressive strength of RAC, while the RCA has the greatest impact. Second, the impact factors of the RRCA, S, W, and SR reached 11.06%, 8.49%, 8.15%, and 6.22%, respectively. It can be seen from the results of the sensitivity analysis that none of the eight input parameters in this study had an impact factor that was too low (lower than 2%). All eight parameters provided useful information for predicting RAC compressive strength.

## 7. Conclusions

RAC is an environmentally friendly construction material with great development potential, and is in line with the concept of sustainable development. With a proper design of mixing proportions, RAC is able to achieve the same performance as ordinary concrete. However due to the impact of the original mortar and cement residual of the old concrete on the recycled aggregate, the compressive strength of RAC is usually weaker than that of ordinary concrete. To guarantee the safe use of RAC, it is necessary to predict the compressive strength of the RAC. In this paper, ANN was applied for the prediction of RAC compressive strength, which verified the usability of the model and allowed the following conclusions to be drawn:

(1) A total of 88 different mix proportions of RAC were designed, and the effects of different water–cement ratios and replacement rates of recycled aggregate regenerated aggregate on RAC compressive strength were studied, with water–cement ratios of 0.35–0.65, and RRCA of 0–100%. The experimental results show that the performance of RAC produced from recycled aggregate can be comparable to that of ordinary concrete. With reasonable mixing proportion design, the RAC compressive strength was able to reach 63 MPa. Under the same water–cement ratio conditions, the RAC slump decreases with increasing RRCA. In addition, the best RRCA rate is 70%.
(2) A total of 840 BPNN models were developed using a trial-and-error approach, for which the C (kg/m^3^), S (kg/m^3^), NCA (kg/m^3^), RCA (kg/m^3^), Water (kg/m^3^) W/C, SR (%), and RRCA (%) were taken as the input parameters. Meanwhile, based on the maximum correlation coefficient R^2^, the optimal BPNN model (8–12–8–1) was selected to predict the RAC compressive strength. The predicted values and the experimental values exhibited good fitting. In addition, the correlation coefficient between the predicted value and the experimental value was 0.96650, and the RMSE reached 2.42.
(3) The sensitivity analysis shows that, all eight of the selected variables was able to greatly affect the compressive strength of RAC; among them, the cement content was the most influential one with respect to its effect on RAC compressive strength. Its impact factor reached 19.78%, while the impact degrees of the other parameters were in the following order: RCA > NCA > W/C > RRCA > S > W > SR.

It can be seen that ANN can help to achieve accurate prediction of RAC compressive strength, and can be applied in other types of concretes. To improve the prediction accuracy, more sample data could be collected during the testing stage, thereby increasing the sample amount of the training set.

## 8. Limitations

This study may not be perfect due to limitations with respect to the authors’ time and knowledge. In practical applications, more sample groups can be set in order to achieve more accurate and reliable prediction.

## Figures and Tables

**Table 1:** Chemical composition and mineral composition of cement.

Composition	Item	Cement (%)
Chemicals	SiO_2_	21.4
	Al_2_O_3_	5.55
	Fe_2_O_3_	3.46
	MgO	1.86
	CaO	64.0
	K_2_O	0.54
	SO_3_	1.42
	Na_2_O	0.26
Compounds	C_3_S	51.0
	C_2_S	23.1
	C_3_A	8.85
	C_4_AF	10.5

**Table 2:** Physical properties of cement.

Density (g/cm^3^)	Fineness (%)	Standard Thick Water Consumption (%)	Set Time (Min)		Compressive Strength (MPa)		Flexural Strength (MPa)		Specific Surface Area (m^2^/kg)
			Initial Setting	Final Set	3 d	28 d	3 d	28 d	
3.32	0.25	26.2	155	215	33.4	49.5	6.7	9.1	360

**Table 3:** Physical properties of the main materials.

Property	NCA	RCA	Sand
Bulk density (g/m^3^)	1.536	1.253	1.758
Apparent density	2.758	2.605	2.765
Stacked porosity (%)	46.5	43.0	38.8
Crush index (%)	11.3	19.6	-
Clay content (%)	0.96	0.26	2.56
Water absorption (%)	0.76	4.88	0.89
Maximum particle size (mm)	25	25	5

**Table 4:** Experimental mixing proportion data.

Division	Grouping	C (kg/m^3^)	S (kg/m^3^)	NCA (kg/m^3^)	RCA (kg/m^3^)	Water (kg/m^3^)	W/C	SR (%)	RRCA (%)
P1	G1	350	532.2	987.2	0	175	0.5	35	0
		350	532	888.4	98.7	175	0.5	35	10
		350	531.7	789.7	197.4	175	0.5	35	20
		350	531.7	691.0	296.1	175	0.5	35	30
		350	531.6	592.3	394.8	175	0.5	35	40
		350	530.8	493.6	493.6	175	0.5	35	50
		350	530.9	394.8	592.3	175	0.5	35	60
		350	531.6	296.1	691.0	175	0.5	35	70
		350	531.3	197.4	789.7	175	0.5	35	80
		350	531.2	98.7	888.4	175	0.5	35	90
		350	530.2	0	988.0	175	0.5	35	100
	G2	350	526.2	974.8	0	192.5	0.55	35	0
		350	525.3	877.3	97.5	192.5	0.55	35	10
		350	523.2	780.0	194.9	192.5	0.55	35	20
		350	523.1	682.3	292.4	192.5	0.55	35	30
		350	523.1	584.8	389.9	192.5	0.55	35	40
		350	521.3	487.4	487.4	192.5	0.55	35	50
		350	522.3	389.9	584.8	192.5	0.55	35	60
		350	522	292.4	682.3	192.5	0.55	35	70
		350	522.3	194.9	779.8	192.5	0.55	35	80
		350	521.3	97.4	877.3	192.5	0.55	35	90
		350	520.3	0	975	192.5	0.55	35	100
	G3	350	517.8	962.2	0	210	0.6	35	0
		350	517.5	866.0	96.2	210	0.6	35	10
		350	516.3	769.7	192.4	210	0.6	35	20
		350	516.2	673.5	288.6	210	0.6	35	30
		350	515.9	577.3	384.8	210	0.6	35	40
		350	515.8	481.1	481.1	210	0.6	35	50
		350	515.4	384.8	577.3	210	0.6	35	60
		350	515.3	288.6	673.5	210	0.6	35	70
		350	515.6	192.4	769.7	210	0.6	35	80
		350	515.2	96.2	865.9	210	0.6	35	90
		350	515.2	0	961.8	210	0.6	35	100
	G4	350	511.2	949.5	0	227.5	0.65	35	0
		350	510.6	855	94.5	227.5	0.65	35	10
		350	510.6	759.6	189.9	227.5	0.65	35	20
		350	510.3	664.6	284.8	227.5	0.65	35	30
		350	510.3	569.7	379.8	227.5	0.65	35	40
		350	509.6	474.7	474.7	227.5	0.65	35	50
		350	509.3	379.8	569.7	227.5	0.65	35	60
		350	508.9	284.8	664.6	227.5	0.65	35	70
		350	508.6	189.9	759.6	227.5	0.65	35	80
		350	508.2	94.9	854.5	227.5	0.65	35	90
		350	508.3	0	950	227.5	0.65	35	100
P2	G5	480	459.3	1070.2	0	153.6	0.32	30	0
		480	459.2	963.2	107	153.6	0.32	30	10
		480	459	856.1	214.0	153.6	0.32	30	20
		480	458.8	749.1	321.0	153.6	0.32	30	30
		480	458.6	642.1	428.0	153.6	0.32	30	40
		480	458.3	535.1	535.1	153.6	0.32	30	50
		480	458.6	428.0	642.1	153.6	0.32	30	60
		480	455.3	321.0	749.1	153.6	0.32	30	70
		480	456.1	214.0	856.1	153.6	0.32	30	80
		480	456.6	107.0	963.1	153.6	0.32	30	90
		480	456.5	0	1071.3	153.6	0.32	30	100
	G6	480	452.8	1509.2	0	177.6	0.37	30	0
		480	452.6	1358.3	150.9	177.6	0.37	30	10
		480	452.9	1207.3	301.8	177.6	0.37	30	20
		480	451.6	1056.4	452.7	177.6	0.37	30	30
		480	451.3	905.5	603.6	177.6	0.37	30	40
		480	451.2	754.6	754.6	177.6	0.37	30	50
		480	450.5	603.6	905.5	177.6	0.37	30	60
		480	450.6	452.7	1056.4	177.6	0.37	30	70
		480	450.3	301.8	1207.3	177.6	0.37	30	80
		480	450.3	150.9	1358.2	177.6	0.37	30	90
		480	450.1	0	1510	177.6	0.37	30	100
	G7	480	447	1042.3	0	201.6	0.42	30	0
		480	447.2	938.1	104.2	201.6	0.42	30	10
		480	447	833.8	208.4	201.6	0.42	30	20
		480	446.9	729.6	312.6	201.6	0.42	30	30
		480	446.8	625.3	416.9	201.6	0.42	30	40
		480	446.5	521.1	521.1	201.6	0.42	30	50
		480	446.5	416.9	625.3	201.6	0.42	30	60
		480	446.1	312.6	729.6	201.6	0.42	30	70
		480	445.8	208.4	833.8	201.6	0.42	30	80
		480	445.3	104.2	938.0	201.6	0.42	30	90
		480	445.6	0	1042	201.6	0.42	30	100
	G8	480	439.5	1025.5	0	230.4	0.47	30	0
		480	439.2	922.9	102.6	230.4	0.47	30	10
		480	439	820.4	205.1	230.4	0.47	30	20
		480	438.6	717.8	307.6	230.4	0.47	30	30
		480	438.6	615.3	410.2	230.4	0.47	30	40
		480	437	512.7	512.7	230.4	0.47	30	50
		480	437.6	410.2	615.3	230.4	0.47	30	60
		480	437	307.6	717.8	230.4	0.47	30	70
		480	436.5	205.1	820.4	230.4	0.47	30	80
		480	436.2	102.5	922.9	230.4	0.47	30	90
		480	436.2	0	1026	230.4	0.47	30	100

**Table 5:** Numerical value statistics of experimental parameters.

Input and Output Parameters	Minimum Value	Maximum	Average	Standard Deviation	Variance
C (kg/m^3^)	350	480	-	-	-
S (kg/m^3^)	436.2	532.2	484.1	36.6	36.9
NCA (kg/m^3^)	0	1509.2	532.6	351.9	353.9
RCA (kg/m^3^)	0	1510	532.6	351.9	354.0
Water (kg/m^3^)	153.6	230.4	196	25	25.1
W/C	0.32	0.65	-	-	
SR (%)	30	35	-	-	
RRCA (%)	0	100	-	-	
CS (MPa)	26	63	41.3	8.7	8.7

**Table 6:** Back propagation neural network (BPNN) model training parameters.

Parameter	Set the Value
Training algorithm	Levenberg–Marquardt Algorithm
Number of hidden layers	1 to 2
Number of hidden layer neurons	1–20
Epochs	500
Performance evaluation	R^2^, RMSE
Transfer function	Log-sigmoid, Tan-sigmoid

**Table 7:** BPNN model ranking based on correlation coefficient R^2^ (TOP10).

The Sorting	Structure	The Transfer Function	R^2^	RMSE
1	8–12–8–1	Log-sigmoid	0.96650	2.42
2	8–16–5–1	Log-sigmoid	0.96236	3.56
3	8–3–5–1	Tan-sigmoid	0.95236	4.56
4	8–15–8–1	Log-sigmoid	0.95233	3.26
5	8–12–1	Tan-sigmoid	0.95016	5.24
6	8–8–6–1	Tan-sigmoid	0.95011	6.35
7	8–13–2–1	Tan-sigmoid	0.94256	2.39
8	8–2–1	Log-sigmoid	0.94026	3.56
9	8–12–9–1	Log-sigmoid	0.94002	3.65
10	8–9–9–1	Log-sigmoid	0.93999	3.2

**Table 8:** Connection weights obtained by optimal BPNN.

Input Parameters								Output Parameter
C	S	NCA	RCA	W	W/C	SR	RRCA	CS
0.825	0.620	0.316	1.265	0.453	0.022	0.822	0.490	−0.370
0.268	0.048	1.256	0.179	0.537	0.767	1.365	0.166	−0.170
0.620	0.316	0.339	0.320	0.560	0.550	0.320	0.220	−0.140
1.256	0.475	0.179	0.580	0.235	0.560	0.250	0.320	−0.730
0.320	0.210	0.360	0.860	0.330	0.240	0.120	0.240	−0.010
0.020	0.320	0.030	0.240	0.120	0.230	0.320	0.010	0.310
0.690	0.120	0.090	0.100	0.120	0.140	0.230	0.210	0.520
0.230	0.360	0.650	0.350	0.320	0.235	0.320	0.210	−0.170
0.050	0.040	0.050	0.180	0.120	0.140	0.120	0.200	0.000
0.320	0.030	0.240	0.120	0.230	0.320	0.010	0.360	−0.110
0.070	0.120	0.000	0.000	0.070	0.120	0.210	0.230	−0.020
0.020	0.001	0.170	0.150	0.210	0.000	0.030	0.020	0.090

## Data Availability

The data presented in this study can be obtained in this paper.
